# Foxtail Millet Ear Detection Method Based on Attention Mechanism and Improved YOLOv5

**DOI:** 10.3390/s22218206

**Published:** 2022-10-26

**Authors:** Shujin Qiu, Yun Li, Huamin Zhao, Xiaobin Li, Xiangyang Yuan

**Affiliations:** 1 College of Agricultural Engineering, Shanxi Agriculture University, Jinzhong 030801, China; 2 College of Agricultural, Shanxi Agricultural University, Jinzhong 030801, China

**Keywords:** foxtail millet ear detection, lightweight, YOLOv5, GhostNet, attention mechanism

## Abstract

In the foxtail millet field, due to the dense distribution of the foxtail millet ears, morphological differences among foxtail millet ears, severe shading of stems and leaves, and complex background, it is difficult to identify the foxtail millet ears. To solve these practical problems, this study proposes a lightweight foxtail millet ear detection method based on improved YOLOv5. The improved model proposes to use the GhostNet module to optimize the model structure of the original YOLOv5, which can reduce the model parameters and the amount of calculation. This study adopts an approach that incorporates the Coordinate Attention (CA) mechanism into the model structure and adjusts the loss function to the Efficient Intersection over Union (EIOU) loss function. Experimental results show that these methods can effectively improve the detection effect of occlusion and small-sized foxtail millet ears. The recall, precision, F_1_ score, and mean Average Precision (mAP) of the improved model were 97.70%, 93.80%, 95.81%, and 96.60%, respectively, the average detection time per image was 0.0181 s, and the model size was 8.12 MB. Comparing the improved model in this study with three lightweight object detection algorithms: YOLOv3_tiny, YOLOv5-Mobilenetv3small, and YOLOv5-Shufflenetv2, the improved model in this study shows better detection performance. It provides technical support to achieve rapid and accurate identification of multiple foxtail millet ear targets in complex environments in the field, which is important for improving foxtail millet ear yield and thus achieving intelligent detection of foxtail millet.

## 1. Introduction

In northern China, foxtail millet is a characteristic miscellaneous grain crop with qualities such as drought and water conservation, a high nutritional value, and environmental friendliness [1]. The demand for cereal production is rising globally due to the need for nutrient-focused agricultural development, green and sustainable ecosystem development, and quality development [2]. The head of foxtail millet is an important indicator to assess the yield and quality of the grain. The detection and research of foxtail millet ear can not only help breeders to accurately evaluate germplasm resources, but also provides agriculturalists with ways to manage production costs at a reduced level. Therefore, it is of great significance to study a foxtail millet ear detection method with a low arithmetic power requirement which can be applied to mobile devices for crop breeding, cultivation, yield improvement, and agricultural production.

In recent years, with the rapid development of computers and artificial intelligence technology, target detection methods based on deep learning have been widely implemented in the field of agriculture [3,4]. In terms of crop organ recognition and detection, researchers have used deep learning models, such as the Faster region-based convolutional neural network (Faster RCNN) [5], You Only Look Once (YOLO) [6,7,8,9], Single Shot MultiBox Detector (SSD) [10], and the Mask region-based convolutional neural network (Mask RCNN) [11], to detect flowers [12,13], stems [14,15], leaves [16,17], and fruits [18,19] have been studied, and certain results have been achieved. For example, a mask R-CNN model fused with an attention mechanism was constructed, which increased the feature extraction capability of the backbone network and could correctly segment apple targets in complex backgrounds [20]. A YOLOv5 model with a lightweight structure was designed, and the squeeze-and-excitation networks were added to the improved backbone network, which can effectively improve the recognition effect of apple-picking robots on occluded apples in complex orchard environment [21]. The introduction of the convolutional block attention module (CBAM) and α-IOU loss function in the YOLOv5 model can improve the recognition of citrus fruits in natural environments [22]. For the ear detection of cereal crops, Zhang et al. proposed a wheat ear detection method based on the attention mechanism pyramid network, which significantly improved the detection effect of occluded wheat ears and smaller wheat ears [23]; Zhang et al. proposed to introduce dilated convolution in the Faster R-CNN model to optimize the Inception_ResNet-v2 feature extraction network, and obtain a rice panicle detection model for different growth stages [24]; Yang et al. proposed to use Faster R-CNN model and SegNet network to detect and segment rice ears, respectively, and obtained a method for extracting rice phenotypic characteristics and predicting ear weight [25]; ZHAO et al. proposed a wheat spike detection method based on the improved YOLOv5 model, which can detect wheat ears in UAV images under occlusion and overlapping conditions [26]. At present, there are few pieces of research on the detection of foxtail millet ears. Only Hao et al. proposed the method of YOLOv4 and adaptive anchor box adjustment to detect foxtail millet ears, but the model still has a large model size [27].

In the above deep learning-based crop organ recognition and detection research, the detection accuracy and speed have been improved, but these deep learning models all rely on high-performance personal computer platforms, which are not suitable for embedded devices with limited computing resources. In order to satisfy the deep learning model in practical production needs, it is required to reduce the deep learning model parameters and reduce the model complexity in the first place. At present, there are two approaches to making deep learning models lightweight: model compression and designing lightweight model structures. For example, Researchers propose to use lightweight models MobileNet v3 and MobileNet v2 to replace the original deep learning model of the backbone feature extraction network design lightweight model [28,29,30]; Wu et al. proposed a lightweight and improved YOLOv3 apple detection model [31]. The improved model uses depthwise separable convolutions to replace ordinary convolutions, and a feature extraction network composed of multiple residual blocks in series. The improved model implements apple detection in the background of complex fruit trees on workstations and Nvidia TX2-embedded development boards. Wu et al. proposed a channel-pruning algorithm to improve the YOLOv4 model, reduce model parameters, model size and inference time, and realize the detection of apple flowers in the natural environment [32]; Yang et al. proposed a fast multi-apple target detection method based on the CenterNet model without anchor boxes, using the lightweight Tiny Hourglass-24 as the backbone network of the model and optimizing the residual module to achieve fast multi-apple targets in dense scenes detection [33]. The light weight of the model structure, however, may reduce target detection accuracy and make it difficult to detect occlusion, adhesion and small-size targets in complex environments. The problem has been addressed in numerous studies by introducing attention mechanisms and multi-scale detection. Li et al. proposed a Yolov4-tiny model for the fast and accurate detection of green peppers [34]. Using technologies such as attention mechanism and adaptive feature fusion of multi-scale detection, the improved model ensures the detection speed of lightweight models and improves the detection performance. Wang et al. proposed an improved Yolov4-tiny model to detect blueberry fruit [35]. Integrating the CBAM attention mechanism into the feature pyramid can ensure the accuracy and speed of blueberry fruit recognition.

In the natural field environment, the morphological differences among foxtail millet ears, overlapping and shading each other and stem and leaf shading are serious, and these factors will increase the difficulty of detection, and the detection accuracy of foxtail millet ears will be low. To solve the above problems, this study proposes a method of foxtail millet ears detection based on attention mechanism and improved YOLOv5. The advantages of this method are as follows: (1) Aiming at the problem of large parameters of the target detection model YOLOv5, the GhostNet module is proposed for lightweight improvement. This method can greatly reduce model parameters and model complexity. (2) In order to improve the detection accuracy of the lightweight YOLOv5 model, this study proposes to integrate the lightweight coordinated attention (CA) mechanism into the backbone feature extraction network. (3) The EIOU loss function is introduced to accelerate the convergence of boundary box loss. The lightweight model proposed in this study can be applied to mobile devices with low computing power to achieve rapid and accurate recognition of multi-spike targets in the field’s natural environment.

## 2. Materials and Methods

### 2.1. Image Acquisition

**Figure 1:**
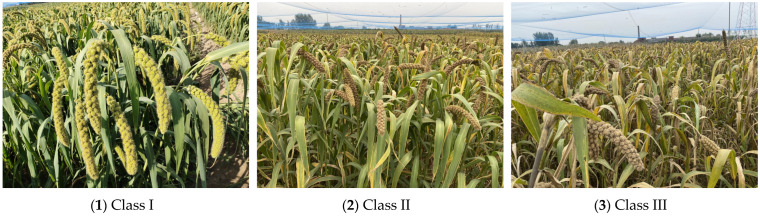
Images of foxtail millet ears at different growth stages.

The original image of the foxtail millet ear was collected at the experimental base in Shen Feng Village, Shanxi Agricultural University. The shape of the ear of the foxtail millet is cylindrical or nearly spinning, and it is mainly in a pendulous state, as shown in Figure 1. In order to obtain more characteristics of the foxtail millet ears, this study was carried out mainly from the top side. A total of 300 original images were collected, and stored in JPG format, including 25 images of the heading stage (Class I), 230 images of the filling stage (Class II), and 45 images of the maturing stage (Class III).

**Figure 2:**
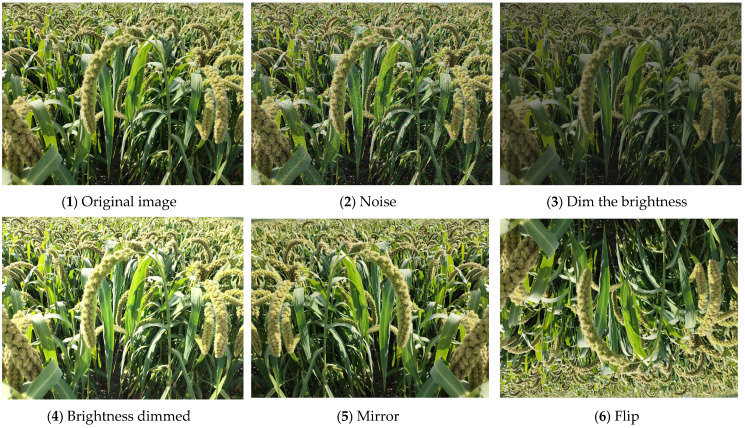
Data augmentation.

Considering the hardware and GPU performance of the laboratory computer, compressing the image pixels to 1024 pixels × 768 pixels and it will help speed up the model training. LabelImg software was used to manually annotate the datasets according to the PASCAL VOC dataset format and the annotation files were saved in XML format. Small datasets may lead to model-fitting phenomena; therefore, data augmentation is used in this study to expand the datasets. The main methods are flipping, mirroring, luminance changes, and adding noise to simulate the conditions that may occur during the capture of images during detection and to improve the generalization ability of the model. The data augmentation results are shown in Figure 2. The original foxtail millet ears dataset was data augmented to obtain a total of 2100 images, which were randomly divided into a training set, a validation set, and a test set according to the ratio of 8:1:1.

### 2.2. Construction of Foxtail Millet Ear Detection Model

#### 2.2.1. YOLO V5

**Figure 3:**
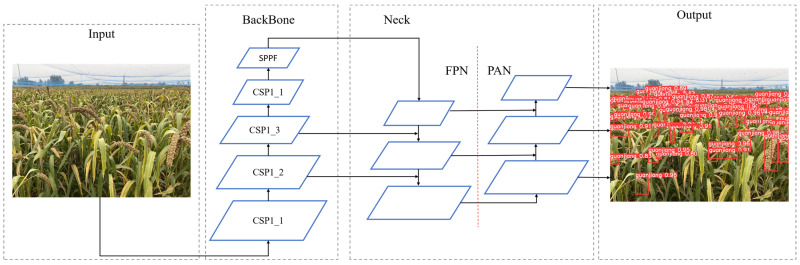
Structure of YOLOv5s.

The YOLOv5 model is a typical one-stage object detection model. It integrates the classification and localization functions of foxtail millet ears into a neural network. The input foxtail millet ear image only needs one network calculation to obtain the location of the target bounding box and target type in the image. As proven by Figure 3, the YOLOv5 model consists of four components of input, backbone, neck, and prediction. According to the distinction in the number of characteristic extraction modules and convolution kernels in the backbone, there are four versions: YOLOv5s, YOLOv5m, YOLOv5l, YOLOv5x., and their model size and parameters progressively increase. In view of the purpose of this research to study the lightweight foxtail millet ear detection model applied in the actual environment; in order to make certain the balance between detection speed, accuracy and model size, YOLOv5s is selected as the basic model for follow-up research.

The input adopts the Mosaic online data augmentation to improve the detection ability of difficult targets, and the adaptive anchor frame can improve the inference speed. The backbone module adopts the CSP1_X structure, where the number of Bottleneck modules determines the depth of the model. The backbone module acts as the backbone feature extraction network for the YOLO v5s model to extract the feature information of the target. The Neck module is formed by the combination of feature pyramid networks(FPN) [36] and Path aggregation network(PAN) [37], which realizes the multi-scale feature fusion function and strengthens the expression ability of target feature information. The Prediction module uses the post-processing NMS algorithm to filter and output multiple prediction boxes generated by the target.

#### 2.2.2. GhostNet

**Figure 4:**
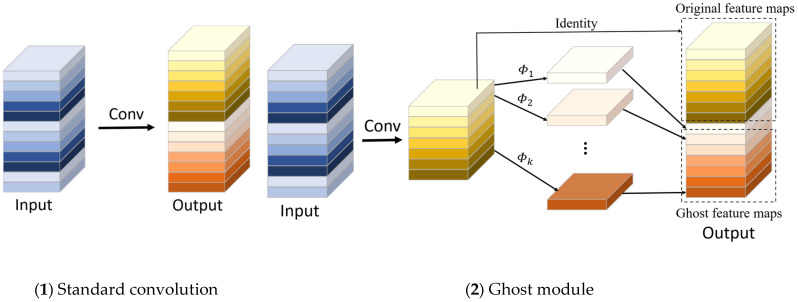
The convolution process of ordinary convolution and GhostConv.

GhostNet is a lightweight feature extraction network proposed by Huawei’s Noah’s Ark Lab in 2020 [38]. It adopts an end-to-end neural network architecture and outperforms MoblieNetv3. The core of GhostNet is the Ghost module. Figure 4 shows the convolution process between the standard convolution and the Ghost module. The Ghost module introduces linear operations instead of partial convolution. Compared with standard convolution, it is divided into two steps: in the first step, standard convolution is used to generate a small number of intrinsic feature maps; in the second step, more Ghost feature maps are obtained with a small number of parameters based on the feature maps generated in the first step using linear operations, such as depth convolution or shift operations. Finally, the feature maps generated by the two steps are merged to obtain the output feature map of the Ghost module. Under the condition that the input and output feature maps have the same size, the calculation amount of the Ghost module is much lower than that of ordinary convolution, which realizes the acquisition of more feature information with less calculation, and does not negatively affect the performance of the model.

**Figure 5:**
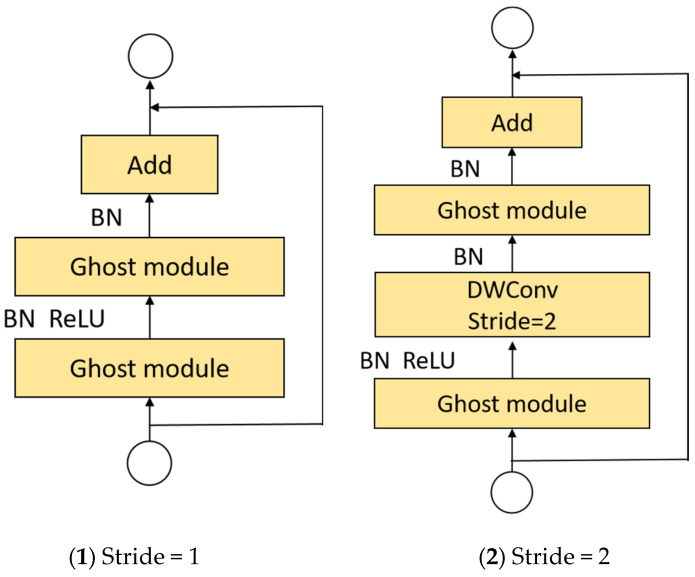
Structure of the Ghost-BottleNeck.

Based on the lightweight advantage of Ghost modules, Ghost-BottleNeck is constructed by stacking two Ghost modules, as shown in Figure 5. When the step size is 1 (Figure 5(1)), the first Ghost module is the expansion layer, expanding the number of channels and increasing the dimensionality of the features; the second Ghost module is used to reduce the number of channels and reduce the dimension of features, and make them match shortcut path. Shortcut connects the input and output of the two Ghost modules. Referring to the structure of MobileNetV2, the ReLU activation function is not used after the second Ghost module, and batch normalization (BN) and ReLU activation functions are introduced after each other layer. When the step size is 2 (Figure 5(2)), the shortcut path consists of a downsampling layer and depthwise convolution with stride = 2, where the depthwise separable convolution can reduce the number of channels.

#### 2.2.3. Coordinate Attention (CA) Mechanism

Foxtail millet in the natural environment of the field, with its dense growth, and the problems of overlapping foxtail millet ears and occlusion of stems and leaves often occur, resulting in the loss of model detection accuracy. To address this problem, this study introduces a lightweight CA mechanism [39] integrated into the backbone feature extraction network of the YOLO V5 model. The attention mechanism helps the model to locate targets of interest more accurately, increase attention to difficult targets such as highly overlapping and obscured targets, suppress natural backgrounds that are not of interest, and improve the accuracy of foxtail millet ears recognition in complex environments.

**Figure 6:**
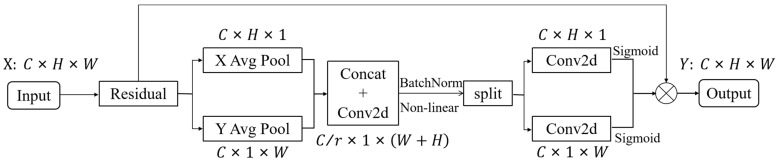
Structure of the CA module.

The CA mechanism is a computing unit that can enhance the feature expression ability of the network. It can take any intermediate feature tensor $X=\left[x_{1},x_{2},\cdots ,x_{C}\right]\in R^{C\times H\times W}$ as input and get an output feature tensor $Y=\left[y_{1},y_{2},\cdots ,y_{C}\right]$ of the same size as the input, *C* represents the number of channels, *H* and *W* denote the height and width of the input feature map, respectively. The CA module consists of two steps: coordinate information embedding and coordinate attention generation, which encodes channel relationships and long-range dependencies through precise location information, and the structure of the CA attention mechanism is shown in Figure 6.

When the size of the input feature map is C×H×W, two pooling kernels of size (H,1) and (1,W) are first used to encode each channel along the horizontal and vertical directions, respectively, aggregating features along the two spatial directions to obtain a pair of direction-aware attention features $Z^{h}$ and $Z^{w}$, which contain distant dependencies in one spatial direction and precise location information in the other. The calculation formula is as follows:

(1) $$Z_{c}^{h}\left(h\right)=\frac{1}{W}\underset{0\leq i<W}{\sum }X_{c}\left(h,i\right)$$

(2) $$Z_{c}^{w}\left(w\right)=\frac{1}{H}\underset{0\leq j<H}{\sum }X_{c}\left(j,w\right)$$

$Z_{c}^{h}\left(h\right)$ denotes the output of the *c*-th channel with height *h*; $Z_{c}^{w}\left(w\right)$ denotes the output of the *c*-th channel with width w; H and W denote the height and width of the input feature map, respectively. The output feature maps from both directions are stitched together and an $F_{1}$ transform is performed in a 1×1 shared convolution kernel to generate an intermediate feature map f:

(3) $$f=\delta \left(F_{1}\left(\left[z^{h},z^{w}\right]\right)\right)$$

$f\in R^{C/r\times \left(H+W\right)}$ is an intermediate feature map containing horizontal and vertical spatial information, *r* is the downsampling ratio, and δ represents the nonlinear activation function. After batchnorm and non-linear activation function processing, the intermediate feature map is sliced into two independent vectors $f^{h}\in R^{C/r\times H}$ and $f^{w}\in R^{C/r\times W}$ along the spatial dimension. Then use two 1×1 convolutions to perform $F_{h}$ and $F_{w}$ transformation and sigmoid function to perform feature transformation, so that the independent tensors $f_{h}$ and $f_{w}$ have the same number of channels as the input feature map, and the output is:

(4) $$g^{h}=\sigma \left(F_{h}\left(f^{h}\right)\right)$$

(5) $$g^{w}=\sigma \left(F_{w}\left(f^{w}\right)\right)$$

where σ denotes the sigmoid activation function. After expanding the output feature tensor $g^{h}$ and $g^{w}$, they are combined into the attention weight matrix. The output tensor $y_{c}$ of the final coordinate attention module is:

(6) $$y_{c}\left(i,j\right)=x_{c}\left(i,j\right)\times g_{c}^{h}\left(i\right)\times g_{c}^{w}\left(j\right)$$

#### 2.2.4. Loss Function Improvement

The loss function part of the target detection model mainly calculates three loss functions viz., bounding box loss, classification loss, and object confidence loss In the YOLO v5 model, CIOU Loss is often used to calculate the loss of the bounding box to make the prediction box more consistent with the true box. The principle of CIOU Loss is as follows:

(7) $$\{\begin{array}{c} Loss_{CIOU}=1-IOU+\frac{\rho^{2}\left(b,b^{gt}\right)}{d^{2}}+\alpha v\\ IOU=\frac{|b\cap b^{gt}|}{|b\cup b^{gt}|}\\ \alpha =\frac{v}{\left(1-IOU\right)+v}\\ \nu =\frac{4}{\pi^{2}}{\left(arctan\frac{w^{gt}}{h^{gt}}-arctan\frac{w}{h}\right)}^{2} \end{array}$$

b and $b^{gt}$ are the center point of the prediction box and the center point of the true box; ρ represents the Euclidean distance between the two center points; α is the weight function; ν represents the variance of the diagonal angle between the true box and the prediction box; *w* and *h* are the height and width of the prediction box; $w^{gt}$ and $h^{gt}$ are the height and width of the target box.

Nevertheless, there are still some problems with CIOU Loss, for example, it does not take into account the difference in the aspect ratio of the bounding box during the regression, that is, it does not truly reflect the relationship between $w^{gt}/h^{gt}$ and w/h [40]. As a result, this study considers the use of EIOU Loss [41] to calculate the loss of bounding boxes. EIOU splits the aspect ratio of the bounding box on the basis of CIOU. EIOU uses the method of calculating the actual error of the target box and the prediction box separately, which makes the model training converge faster. The expression of EIOU Loss is as follows:

(8) $$Loss_{EIOU}=1-IOU+\frac{\rho^{2}\left(b,b^{gt}\right)}{d^{2}}+\frac{\rho^{2}\left(w,w^{gt}\right)}{d_{w}^{2}}+\frac{\rho^{2}\left(h,h^{gt}\right)}{d_{h}^{2}}$$

where $d_{w}$ and $d_{h}$ are the width and height of the smallest closed box covering the ground-truth box of the predicted box.

### 2.3. Improved Model

**Figure 7:**
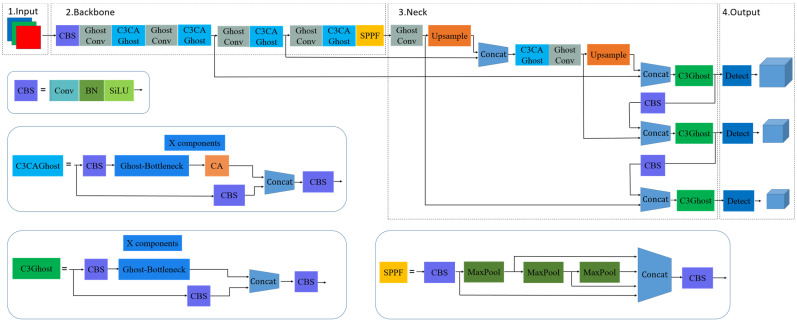
Structure of the improved YOLOv5 model.

The structure diagram of the improved YOLOv5 model is shown in Figure 7, which can be used for real-time detection of foxtail millet ears in the field. The lightweight GhostNet algorithm is used to improve the Yolo V5s model by reducing the size of the model and the number of parameters, effectively saving computational resources. Considering the factors such as dense growth of foxtail millet ears, inconsistent scale, and serious shading in complex field environments, it is easy to cause the loss of target information, which is not conducive to the detection of foxtail millet ears in the field. This study introduces the CA attention mechanism, which adds position information to the channel attention and helps the lightweight model to obtain more feature information. The CA attention mechanism is incorporated into Backbone’s Ghost-BottleNeck structure to reconstitute the backbone feature extraction network of the lightweight model, giving it a strong feature extraction capability without adding redundant network computations.

## 3. Results and Discussion

This study is based on the improvement of the YOLOv5s model, and the experimental run environment is shown in Table 1.

**Figure 8:**
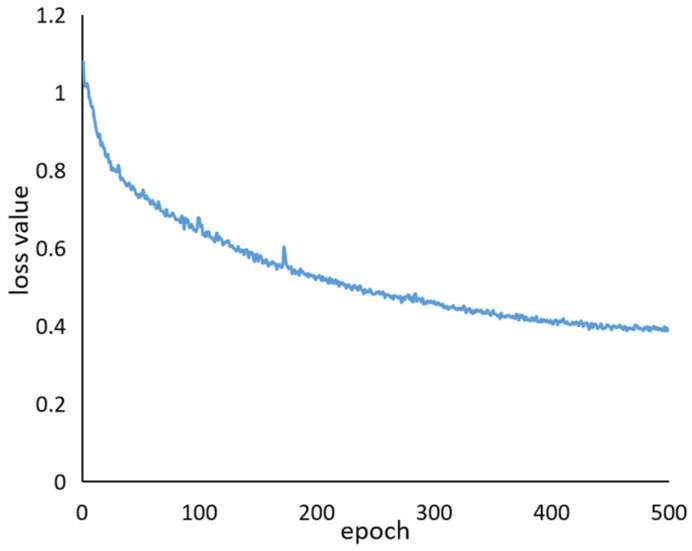
Training loss curve for the improved model.

The batch size of the model is four, and the number of iterations is set to 500. The initial learning rate is 0.01. The loss values of the model training are shown in Figure 8. During the first 200 rounds of training, the loss value drops sharply, and when it is close to 450 times, the model converges. Therefore, in this study, the model output after 500 rounds of training is determined as the foxtail millet ear detection model.

### 3.1. Evaluation Indicators

In this study, the precision (*P*), recall (*R*), mean Average Precision (*mAP*), and $F_{1}$ score were used to evaluate the detection performance of the model. The specific calculation is as follows:

(9) $$Precision=\frac{TP}{TP+FP}\times 100\%$$

(10) $$Recall=\frac{TP}{TP+FN}\times 100\%$$

(11) $$mAP=\frac{1}{C}\overset{N}{\underset{M=i}{\sum }}P\left(k\right)\Delta R\left(k\right)$$

(12) $$F_{1}=\frac{2\times Precision\times Recall}{Precision+Recall}$$

where TP is the number of correctly identified ears of foxtail millet; FP is the number of incorrectly or unidentified ears of foxtail millet; and FN is the number of incorrectly identified ears of foxtail millet targets. C is the number of categories of foxtail millet ears; M and N represent the number of IOU thresholds and IOU thresholds; P(k) and R(k) are the precision and recall rates.

The F_1_ score is a harmonic average sum of precision and recall. Parameters and floating-point operations (FLOPs) are used to measure the network complexity of the model, with smaller values indicating a lower network complexity of the model.

### 3.2. Comparison of Attentional Mechanism Fusion Positions

To obtain the best detection model, the CA module was fused to different positions of the model and the influence of the CA module on the YOLOv5s-Ghost model when in different positions was investigated. The CA module was incorporated into the Ghost bottleneck of the backbone and neck sections of the lightweight model YOLOv5s-Ghost to generate three new models and the test results are shown in Table 2. The backbone part is the backbone feature extraction network of the model, which is the key part for information extraction from the input feature map. Difficult target features such as small size and severe occlusion will be overlooked during the extraction process, resulting in information loss. The addition of the CA module helps the model to enhance the attention and localization of this part of the target, suppressing uninteresting targets and reducing the loss of foxtail millet ear feature information during feature extraction.

### 3.3. Ablation Experiments

To verify the validity of the improved model in this study, this study used the self-built foxtail millet ear dataset for ablation experiments. The specific results of the ablation experiments are shown in Table 3. “√” meant to use corresponding methods to improve the model, and “-“ meant not to use corresponding methods. The model size of the original YOLOv5s model is 10.45 MB. Using the lightweight YOLOv5s-Ghost model of the GhostNet module, the size of the model is reduced to 7.45 MB. The parameters and floating-point operations are also reduced to varying degrees. It shows that the improved method using GhostNet algorithm has a lightweight effect on the YOLOv5s model. When using the CA module alone in the model, the mAP of the YOLOv5s model increases from 96.4% to 96.5%; the mAP of the YOLOv5s-Ghost model increases from 94.6% to 95.9%. The results show that the CA module can improve the feature extraction ability of the model backbone feature extraction network, can add more attention to the objects of interest, and suppress the information of useless objects.

The detection accuracy of YOLOv5s model and YOLOv5s-Ghost model using the CA module alone is 0.1% and 1.3% higher than that of YOLOv5s model and YOLOv5s-Ghost model without the CA module, respectively. The results show that the CA module can improve the feature extraction ability of the model backbone feature extraction network, can add more attention to the objects of interest, and suppress the information of useless objects. The detection model using the CA module can improve the model detection performance. The EIOU loss function is used to reduce the loss of bounding boxes during regression. When the CA module and EIOU loss function are applied simultaneously, the performance of the model is improved in all aspects. By applying both the CA module and the EIOU loss function to the model for improvement, the performance of the model has improved in all aspects. The F_1_ scores of the YOLOv5s model and the YOLOv5s-Ghost model increased by 0.17% and 3.00%, respectively. The mAP and F_1_ scores of the improved model in this study are 96.60% and 95.38%, which are 0.2% and 0.43% higher than the original YOLOv5s model. The model size is 8.12 MB, which is 2.33 MB lower than the original YOLOv5s model, and the parameters and floating-point operations are also significantly reduced. The results show that although the detection time of the improved model increases, the complexity of the model decreases significantly while maintaining better detection accuracy.

**Figure 9:**
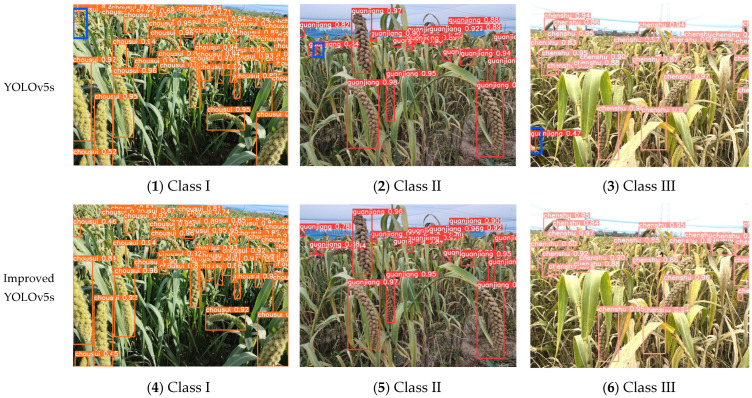
Detection of foxtail millet ears at different periods by the improved YOLOv5s model and the original YOLOv5s model.

Figure 9 shows the visualization results of the improved YOLOv5s model and the original YOLOv5s model for the detection of foxtail millet ears in three growth stages. As can be seen from the figure, the improved YOLOv5s model is close to the same as the original YOLOv5s model in terms of detection, and they can both achieve detection for the front row of foxtail millet ears. However, the YOLOv5s model still suffers from missed and false detections, such as those marked by the blue boxes in Figure 9(1–3). The experimental results show that the improved YOLOv5s model in this research has obvious advantages in detecting difficult samples.

### 3.4. Model Performance Comparison

The YOLOv5s model improved by the lightweight network Mobilenetv3small and Shufflenetv2 and the lightweight model YOLOV3_tiny model were compared with the improved model in this study under the same configuration environment. The results are shown in Table 4. On the same dataset, the F_1_ score of YOLOV3_tiny, YOLOv5-Mobilenetv3small, and YOLOv5-shufflenetv2 were 77.17%, 86.36% and 88.64%, respectively, while the F_1_ score of the improved YOLOv5s model in this study was 95.81%, which was higher than that of YOLOV3_tiny, YOLOv5-mobilenetv3small and YOLOv5-Shufflenetv2 by 18.64%, 9.45%, and 7.17%, respectively. In terms of detection accuracy, the mAP of the improved YOLOv5s is 96.6%, which is higher than that of YOLOv3_tiny, YOLOv5-Mobilenetv3small and YOLOv5-Shufflenetv2. In respect of time, the detection time of the improved YOLOv5s for each image has slightly higher than that of the other three models, achieving good detection accuracy at the expense of losing less is detection time. Considering the detection accuracy and detection speed comprehensively, the improved YOLOv5s is more suitable for completing the detection task of foxtail millet ears in complex field environments.

## 4. Conclusions

This paper proposed an improved lightweight model to detect foxtail millet ears in complex field environments. The traditional lightweight detection model pair used for identifying difficult samples of foxtail millet ears, such as small foxtail millet ears, highly dense foxtail millet ears, and shaded foxtail millet ears had low accuracy and poor robustness. In this research, we established a foxtail millet ear detection model based on an attention mechanism and lightweight improved YOLOv5. In the improved model architecture, to realize the lightweight improvement of the model, the original YOLOv5 model was improved by using the lightweight GhostNet module. To identify blocked foxtail millet ears and dense foxtail millet ears, the CA module was fused into the Ghost-BottleNeck module in the backbone structure, and the EIOU loss function was introduced to accelerate the convergence of the bounding box. According to the proposed improved model, the recall, precision, mAP and F_1_ scores of the improved model were 97.90%, 93.80%, 96.60%, and 95.81%, respectively. The model size was 8.12 MB, and the average detection time per image reached 0.0181 s. The experimental results showed that the improved YOLOv5 model can effectively improve the detection effect of difficult samples while ensuring the model size and detection speed of the lightweight model. The parameters and floating-point operations of the improved YOLOv5 model were reduced by 24.51% and 34.72% compared with the original YOLOv5s model. The improved YOLOv5s model was compared to three lightweight models, YOLOv3_tiny, YOLOv5-Mobilenetv3small, and YOLOv5-Shufflenetv2, which had the highest mean accuracy and a faster mean detection time of 0.0181 s. Therefore, this research provides a new idea for intelligent monitoring and automated harvesting of foxtail millet ear growth, and has a positive impact on the scientific and intelligent agricultural production activities of agricultural workers.

## Figures and Tables

**Table 1:** Experimental Environment.

Configuration	Parameter
CPU	AMD Ryzen 7 5800H
GPU	6 GB NVIDIA GeForce RTX 3060 Latop
Accelerated environment	CUDA11.4 CUDNN 8.2.4
Development environment	Pycharm2021.3
Operating system	Windows 10

**Table 2:** Comparison of CA module fusion results.

Models	P/%	R/%	mAP/%	F_1_/%	Parameters/10^6^	Floating Point FLOPs/10^9^	Model Size/MB
YOLOv5s-Ghost + EIOU	94.60	92.40	95.70	93.50	3.80	8.20	7.45
YOLOv5s-Ghost + CA-backbone + EIOU (Improved YOLOv5s)	97.90	93.80	96.60	95.81	3.99	9.40	8.12
YOLOv5s-Ghost + CA-neck + EIOU	95.80	89.20	94.90	92.40	3.60	8.00	7.29
YOLOv5s-Ghost + CA-all + EIOU	96.50	90.30	95.60	93.30	3.90	9.30	7.95

**Table 3:** Ablation experiments.

GhostNet	CA	EIOU	AP/%			F_1_/%	mAP/%	Average Detection Time per Image/s	Parameters/10^6^	FLOPs/10^9^	Model Size/MB
			Class I	Class II	Class III						
-	-	-	98.40	97.00	94.00	95.38	96.40	0.0146	5.33	14.40	10.45
-	√	-	98.20	97.30	94.10	95.21	96.50	0.0165	5.25	10.90	10.38
-	-	√	97.70	97.40	94.00	95.07	96.40	0.0169	5.30	14.40	10.45
-	√	√	98.70	97.60	94.10	95.55	96.80	0.0154	5.25	10.90	10.37
√	-	-	96.60	94.90	92.20	92.81	94.60	0.0148	3.68	8.10	7.45
√	√	-	97.80	96.00	93.80	94.54	95.90	0.0180	3.99	9.40	8.12
√	-	√	97.00	95.00	95.20	93.49	95.70	0.0154	3.75	8.20	7.45
√	√	√	98.00	97.70	94.10	95.81	96.60	0.0181	3.99	9.40	8.12

**Table 4:** Comparison of detection capabilities of different network models.

Model	AP/%			mAP/%	F_1_/%	Average Detection Time per Image/s
	Class I	Class II	Class III			
Improved YOLOv5s	98.00	97.70	94.10	96.60	95.81	0.0181
YOLOv3_tiny	77.60	75.70	81.60	78.30	77.17	0.0090
YOLOv5-Mobilenetv3small	92.90	87.00	87.40	89.10	86.36	0.0175
YOLOv5-Shufflenetv2	91.00	90.00	90.20	90.40	88.64	0.0152

## Data Availability

Not applicable.
